# Rapamycin treatment of Mandibuloacral Dysplasia cells rescues localization of chromatin-associated proteins and cell cycle dynamics

**DOI:** 10.18632/aging.100680

**Published:** 2014-07-19

**Authors:** Vittoria Cenni, Cristina Capanni, Elisabetta Mattioli, Marta Columbaro, Manfred Wehnert, Michela Ortolani, Milena Fini, Giuseppe Novelli, Jessika Bertacchini, Nadir M. Maraldi, Sandra Marmiroli, Maria Rosaria D'Apice, Sabino Prencipe, Stefano Squarzoni, Giovanna Lattanzi

**Affiliations:** ^1^ National Research Council of Italy, Institute of Molecular Genetics, IGM-CNR-IOR, Bologna, Italy; ^2^ Rizzoli Orthopedic Institute, Laboratory of Musculoskeletal Cell Biology, Bologna, Italy; ^3^ Institute of Human Genetics, University of Greifswald, Germany; ^4^ Rizzoli Orthopedic Institute, Laboratory of Preclinical and Surgical Studies and BITTA, RIT, Bologna, Italy; ^5^ Department of Biomedicine and Prevention, Tor Vergata University, Rome, Italy; ^6^ Department of Laboratory, CEIA, University of Modena and Reggio Emilia, Modena, Italy; ^7^ Fondazione Policlinico Tor Vergata, Rome, Italy

**Keywords:** Mandibuloacral Dysplasia (MADA), Prelamin A, SIRT-1, Oct-1, Rapamycin

## Abstract

Lamin A is a key component of the nuclear lamina produced through post-translational processing of its precursor known as prelamin A. *LMNA* mutations leading to farnesylated prelamin A accumulation are known to cause lipodystrophy, progeroid and developmental diseases, including Mandibuloacral dysplasia, a mild progeroid syndrome with partial lipodystrophy and altered bone turnover. Thus, degradation of prelamin A is expected to improve the disease phenotype. Here, we show different susceptibilities of prelamin A forms to proteolysis and further demonstrate that treatment with rapamycin efficiently and selectively triggers lysosomal degradation of farnesylated prelamin A, the most toxic processing intermediate. Importantly, rapamycin treatment of Mandibuloacral dysplasia cells, which feature very low levels of the NAD-dependent sirtuin SIRT-1 in the nuclear matrix, restores SIRT-1 localization and distribution of chromatin markers, elicits release of the transcription factor Oct-1 and determines shortening of the prolonged S-phase. These findings indicate the drug as a possible treatment for Mandibuloacral dysplasia.

## INTRODUCTION

Prelamin A is the major splicing product of the *LMNA* gene, which undergoes complex and rapid post-translational modifications yielding mature lamin A [1]. Four processing intermediates, including farnesylated prelamin A, are produced in the lamin A maturation pathway. Prelamin A plays a physiological role in muscle, but requires fine tuning during differentiation [2],[3] to accomplish its biological role in the regulation of nuclear envelope-mediated chromatin remodeling and myonuclear positioning. Moreover, sub-toxic amounts of prelamin A are expressed in cells during physiological ageing [4]. On the other hand, high levels of farnesylated prelamin A are toxic to cells, leading to nuclear envelope folding, chromatin damage and cellular senescence, such as in vascular smooth muscle cells [5], and represent the major hallmark of syndromic laminopathies associated or not with premature ageing [6]. Accumulation of toxic amounts of prelamin A, either due to *LMNA* mutations, or due to mutation of the prelamin A endoprotease ZMPSTE24, which catalyzes protein maturation, is the molecular basis of Hutchinson-Gilford progeria syndrome (HGPS), Mandibuloacral dysplasia with accelerated ageing and type A (MADA, OMIM #248370) or type B lipodystrophy (MADB) and Restrictive Dermopathy (RD, OMIM #275210), a severe developmental disorder [6, 7] [8, 9]. RD cells feature accumulation of prelamin A and complete absence of mature lamin A, due to homozygous mutations of the *FACE1* gene, which impair the activity of the prelamin A endoprotease ZMPSTE24 [9, 10]. Accumulation of prelamin A at lower levels occurs in MADA [11] and it has been associated with recruitment of the adipocyte transcription factor SREBP1 in the nuclear periphery and impaired nuclear transactivation activity [12]. Analogous mechanisms of transcription factor sequestration at the nuclear rim have been reported for cFos, which associates with mature lamin A [13], Sp1, which binds prelamin A [14] and Oct-1, which is retained by lamin B1 [15].

Here, we address different susceptibilities of prelamin A forms to proteolysis and demonstrate that treatment with rapamycin efficiently and selectively triggers lysosomal degradation of farnesylated prelamin A and rescues nuclear defects observed in laminopathic cells. In the reported study we observed that MADA fibroblasts and, to a lesser extent RD cells, accumulate Oct-1 in the nuclear envelope and in nucleoplasmic aggregates, while show extremely low levels of LAP2alpha and of the NAD dependent sirtuin SIRT-1 in the nuclear matrix. Oct-1 recruitment in MADA or RD cells is not associated with lamin B1 accumulation [15]. Instead prelamin A, and mostly its R527H mutated isoform found in MADA, is able to sequester Oct-1. Importantly, rapamycin, previously shown to reduce truncated prelamin A levels in HGPS [16], elicits re-localization of LAP2alpha and Oct-1, suggesting the rescue of chromatin dynamics [15, 17]. While proliferation activity is slightly affected by drug treatment, the ultimate effect of rapamycin in MADA cells is the recovery of prolonged S-phase. Here, we suggest that Oct-1 recruitment to damaged DNA sites and PCNA increase facilitate DNA repair and shorten S-phase, ultimately improving chromatin dynamics. These data indicate rapamycin as a suitable drug to be tested for MADA therapy.

## RESULTS

### Regulation of prelamin A degradation

**Figure 1:**
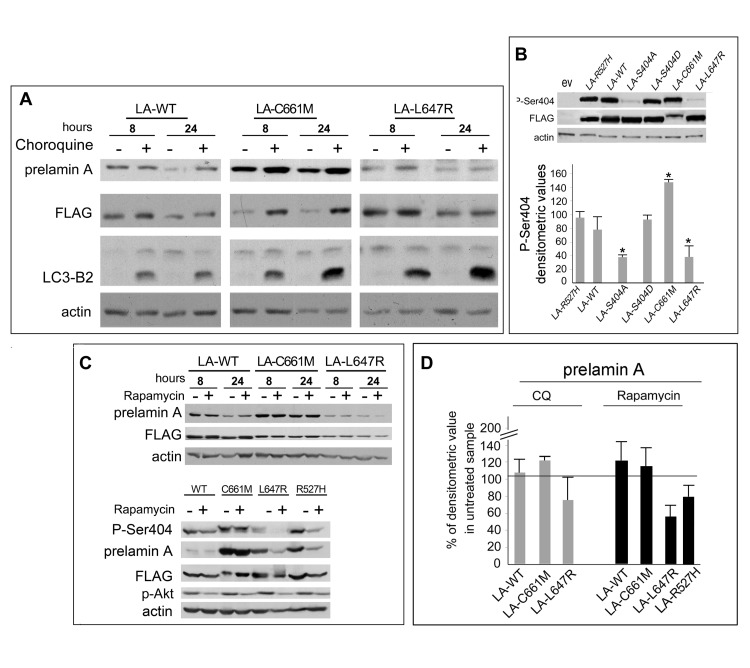
Mechanisms of prelamin A degradation HEK293 cells were transiently transfected with prelamin A constructs yielding wild-type lamin A (LA-WT), non-farnesylatable unprocessable prelamin A (LA-C661M), or farnesylated (partially processed) prelamin A (LA-L647R). (**A**) Transfected cells were subjected to Chloroquine diphosphate (chloroquine) to block lysosome-mediated degradation. Proteins separated on 8% SDS-PAGE were subjected to Western blot analysis. LA-C661M was accumulated in chloroquine-treated cells, indicating lysosomal degradation of non-farnesylated prelamin A. The presence of LC3-B2 band indicates activation of the lysosomal degradation pathway. (**B**) Phosphorylation of Serine 404 (P-Ser404) is detected in LA-WT and LA-C661M, while LA-L647R is minimally phosphorylated. LA-S404A is a non-phosphorylatable lamin A mutant and was tested as a negative control for anti-P-Ser404 antibody, LA-S404D is a phosphomimetic mutant for P-Ser404. Densitometric values of P-Ser404 labeled bands normalized to FLAG-labeled bands are reported in the graph as mean values of triplicate experiments +/− Standard Deviation. Asterisks indicate significantly different values relative to LA-WT (p< 0.05) determined by Student's T test. (**C**) To test rapamycin activity on prelamin A mutants, cells were treated with rapamycin (rapamycin) for the indicated time periods. Farnesylated prelamin A (LA-L647R) was selectively degraded (upper panel). Phosphorylation of Serine 404 (P-Ser404) is shown in the lower panel. (**D**) The densitometric values of anti-prelamin A labeled bands detected in chloroquine or rapamycin-treated cellular lysates were measured. Data are reported as percentage of the densitometric value of each corresponding untreated sample (designated as 100%). The mean values of triplicate experiments +/− Standard Deviation are reported. Actin has been labeled as a loading control. Prelamin A was detected by anti-prelamin A (Santa Cruz Sc-6214) antibody or by anti- FLAG antibody (Sigma-M2).

The toxic molecule in progeroid and developmental laminopathies is prelamin A, which is subjected to rapid and modulated processing in healthy human cells [18], while it is accumulated to toxic levels in HGPS, MADA and RD, as well as in Dunningan-type familial partial lipodystrophy [6, 8-10, 19]. Activation of autophagy has been reported in laminopathic mouse models, as a mechanism aimed at reducing the toxic effects triggered by mutated lamins [20, 21]. A similar detoxification activity has been reported for autophagy in other inherited disease models [22]. To test the susceptibility of prelamin A to lysosomal degradation, we decided to block the cellular autophagic activity in human cells by chloroquine (CQ) and check whether prelamin A was accumulated [23]. HEK293 cells were transfected with different prelamin A constructs [24], and treated at different times with CQ. As shown in Fig. 1A, non-farnesylated prelamin A, produced by the LA-C661M mutant, accumulated in cells exposed to CQ. This was not the case of prelamin A obtained following the transfection of LA-WT and LA-L647R, i.e. in cells expressing processable prelamin A (LA-WT) or farnesylated prelamin A (LA-L647R) (Fig. 1A). Thus, lysosomal degradation appeared to be impaired in the presence of the farnesylated residue at the prelamin A CaaX box, while non-farnesylated prelamin A appeared to be degraded through the lysosomal pathway [3, 23]. We have recently identified a phosphorylation-dependent mechanism of prelamin A degradation activated by the lamin A kinase AKT1, which catalyses phosphorylation of prelamin A Serine 404 [25-27]. Data reported in Figure 1B show that non-farnesylated prelamin A, which undergoes spontaneous degradation, is phosphorylated at Serine 404, while farnesylated prelamin A is minimally phosphorylated. Thus, phosphorylation of non-farnesylated prelamin A may in part explain its susceptibility to spontaneous degradation, while minimal phosphorylation of farnesylated prelamin A may confer stability to the protein isoform [27]. Importantly, mutated R527H prelamin A, which is expressed at the homozygous state in MADA, is also phosphorylated at Serine 404 (Figure 1B).

### Rapamycin effects on prelamin A mutants

Autophagy is negatively regulated by mTOR, the mammalian target of rapamycin [28]. As an inhibitor of the mTOR pathway, rapamycin can therefore induce autophagy in all mammalian cell types. Thus, we decided to check whether rapamycin might accelerate prelamin A degradation. HEK293 cells expressing LA-WT, LA-C661M or LA-L647R prelamin A were treated with rapamycin and lysates tested for the prelamin A amount (Fig. 1C). Strikingly, rapamycin was able to induce degradation of farnesylated prelamin A, as determined by immunoblot analysis (Fig. 1C, upper panel). In fact, the best target of rapamycin was LA-L647R, i.e. the farnesylated form of prelamin A [1, 6], while the non-farnesylated isoform (LA-C661M) or mature lamin A (LA-WT) appeared to be accumulated following rapamycin treatment (Fig. 1C). Thus, we concluded that the farnesylated C-terminus of prelamin A favors protein targeting to degradation in rapamycin-treated cells. Moreover, although rapamycin is known to inactivate mTOR, which lies downstream of AKT1 [29], we observed some inhibitory activity of the drug on AKT1 (Fig. 1C, lower panel), likely due to further effects on TORC2, which phosphorylates AKT1 [30].

The latter finding can in part explain lack of prelamin A degradation in cells expressing LA-WT and LA-C661M, since these prelamin A forms require AKT1 activity for degradation, as shown above. Importantly, rapamycin was able to reduce the levels of both LA-L647R and farnesylated LA-R527H prelamin A, which is accumulated in MADA (Fig. 1C) [19]. Thus, we suspected that rapamycin treatment of laminopathic cells could reduce farnesylated prelamin A accumulation and downstream pathogenetic effects, as previously observed for progerin in HGPS cells [16, 31].

### Rapamycin treatment of laminopathic fibroblasts

To test this hypothesis, we first analyzed prelamin A degradation pathways in RD or MADA cells. Moreover, in order to test rapamycin effect on prelamin A, we treated cultured laminopathic fibroblasts with rapamycin for 4 days.

**Figure 2:**
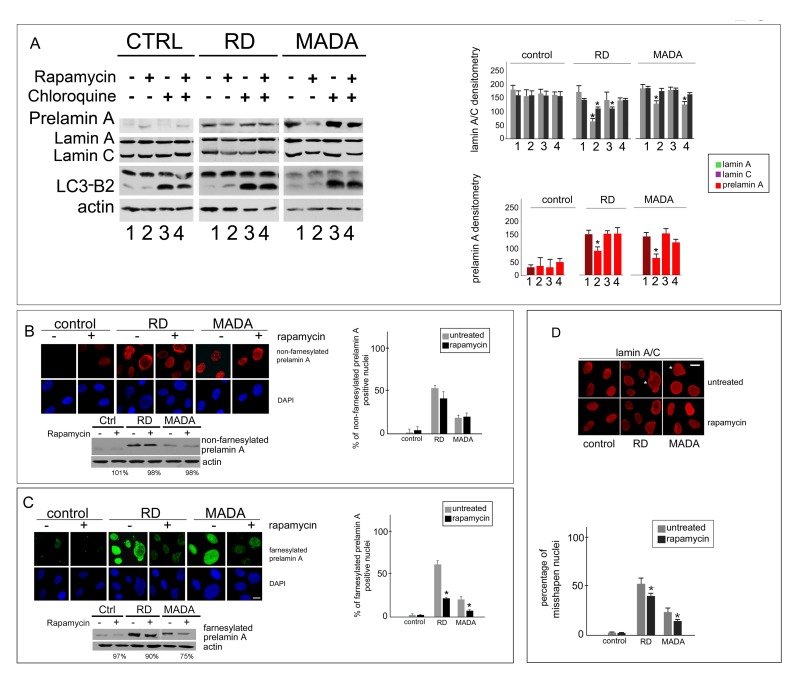
Prelamin A degradation in RD and MADA cells. (**A**) Cellular lysates from control, RD or MADA fibroblasts were subjected to Western blot analysis to test prelamin A, lamin A, lamin C and LC3B2 levels under different experimental conditions. Actin was labeled as a loading control. Cultured fibroblasts were left untreated or treated with rapamycin and/or chloroquine. Densitometric analysis of lamin A, lamin C and prelamin A immunoblotted bands is shown (dark red bars highlight untreated samples). Statistically significant differences (p<0.05), measured by the Mann-Whitney test, are indicated by asterisks. Prelamin A was detected by anti-prelamin A antibody (Santa Cruz Sc-6214), lamin A/C by anti-lamin A/C antibody (Santa Cruz Sc-6215). (**B**) Prelamin A immunofluorescence staining performed using anti-prelamin A 1188-1 antibody, which labels non-farnesylated prelamin A, was detected using a TRITC-conjugated secondary antibody (red). (**C**) Prelamin A immunofluorescence staining performed using anti-prelamin A 1188-2 antibody, which labels farnesylated prelamin A, was detected using a FITC-conjugated secondary antibody (green). Control, RD or MADA fibroblasts were left untreated (−) or treated with rapamycin (+) for 4 days. Bar, 10 μm. Statistical evaluation of non-farnesylated and farnesylated prelamin A-labeled nuclei is reported in the graphs in (**B**) and (**C**), respectively. 200 nuclei per sample were counted. Western blots showing non farnesylated and farnesylated prelamin A on the same treated or untreated cultures are also shown in (**B**) and (**C**), respectively. Densitometric values of prelamin A bands in rapamycin-treated samples are reported below each immunoblotted band as percentage of the intensity detected in the corresponding untreated sample. (**D**) Lamin A/C staining of control, RD and MADA fibroblasts showing misshapen nuclei in laminopathic cells left untreated (untreated) or subjected to 4 day rapamycin treatment (rapamycin). The quantitative analysis is reported in the graph. Statistically significant differences (p<0.05) relative to untreated corresponding samples were calculated using the Mann-Whitney non-parametric test and are indicated by asterisks.

Conversion of microtubule-associated protein 1 light chain 3 (LC3), from the cytosolic LC3-B to the autophagosome-associated LC3-B2 form [23, 32] was observed in untreated samples, indicating some activation of autophagy in cells even under basal conditions (Fig. 2A). However, the highest rate of LC3-B2 to LC3B band intensity was observed in MADA, indicating an active autophagic process (Fig. 2A). Consistent with this observation, treatment with CQ did not elicit accumulation of prelamin A in control or RD samples, but prelamin A was accumulated in chloroquine-treated MADA cells (Fig. 2A).

In rapamycin-treated RD cells, prelamin A level was reduced, although in control fibroblasts, rapamycin did not significantly modify the level of lamin A and slightly increased prelamin A (Fig. 2A). In MADA fibroblasts, both mature lamin A and prelamin A are detected (Fig. 2A), all the A-type lamins produced in those cells bearing the R527H homozygote mutation [19, 33]. Moreover, different prelamin A forms are accumulated [11]. In rapamycin-treated MADA fibroblasts, levels of non farnesylated prelamin A were slightly affected, while farnesylated prelamin A was significantly decreased (Fig. 2B and C). Similar effect of rapamycin on prelamin A forms was obtained in RD cells (Fig. 2B and C).

The whole evaluation of the results obtained in LA-R527H prelamin A expressing cells (Fig. 1C and Fig. 2A and C) suggested that phosphorylation and hence degradation of mature lamin A and non-farnesylated prelamin A occurs in MADA, while farnesylated LA-R527H prelamin A is accumulated and can be reduced by rapamycin treatment. Analysis of nuclear abnormalities considering blebs, honeycomb structures and enlarged nuclei as a whole, showed that the percentage of misshapen nuclei dropped from 20% to 10% in rapamycin-treated MADA fibroblasts (Fig. 2D), suggesting rescue of pathogenetic pathways.

### Mechanisms regulating prelamin A turnover

We further hypothesized that rapamycin could influence *LMNA* expression in RD and/or MADA. Quantitative RT-PCR analysis showed that *LMNA* expression was two folds increased in untreated MADA cells relative to controls and it was slightly reduced by rapamycin treatment (Fig. S1). This effect could be related to the slight reduction of AKT1 activity exerted by rapamycin (see above), since AKT1 also affects *LMNA* expression [27]. Importantly, different fate of spliced mRNAs for prelamin A and lamin C was observed. Prelamin A mRNA was downregulated in RD and MADA, while lamin C was upregulated (Fig. S1). Rapamycin reduced lamin C levels in any sample, but increased prelamin A mRNA expression in MADA (Fig. S1). Moreover, in rapamycin-treated fibroblasts *FACE1* mRNA expression was slightly affected (Fig. S1). The rate between *LMNA* and *FACE1* expression was not changed by drug administration, supporting the view that endoprotease expression is related to *LMNA* levels.

Thus, we concluded that rapamycin acts on prelamin A, in RD and MADA, at both the transcriptional and post-translational level, but relevant changes are due to protein degradation.

### Rescue of LAP2alpha and Oct-1 localization in rapamycin-treated laminopathic fibroblasts

**Figure 3:**
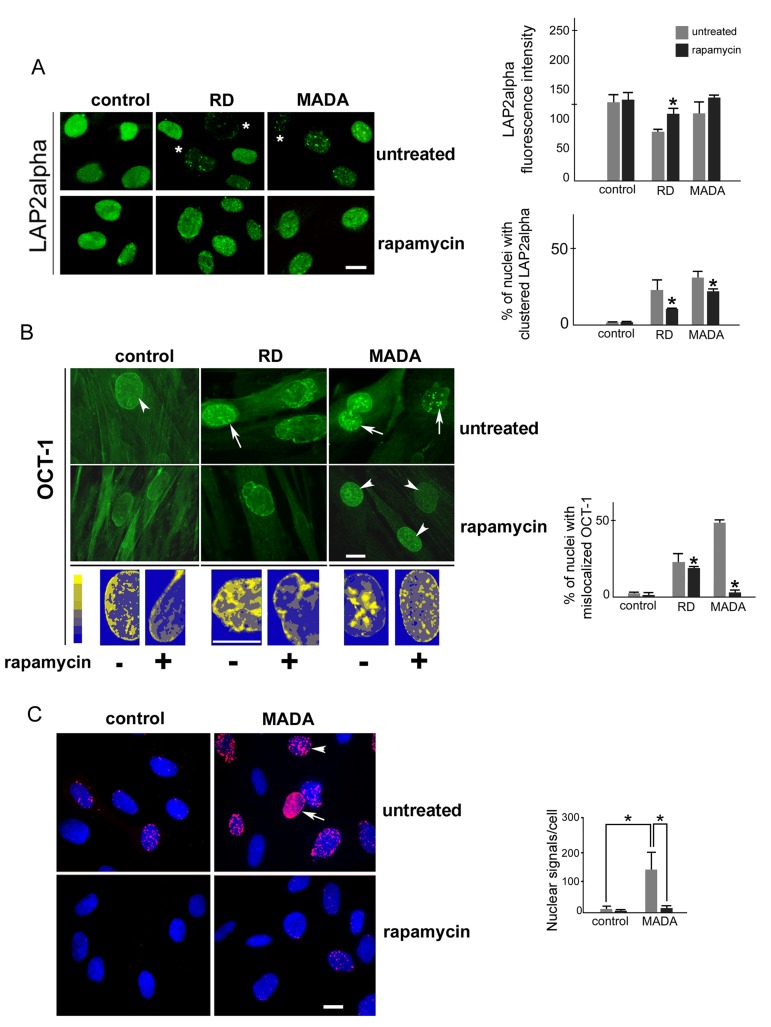
LAP2alpha and Oct-1 localization are affected in MADA and rescued by rapamycin (**A**) Control, RD and MADA cells left untreated (untreated) or after rapamycin treatment (rapamycin) were stained for LAP2alpha. Levels of LAP2alpha fluorescence intensity and the percentage of nuclei with LAP2alpha mislocalized in nuclear clusters are reported in the upper and lower graph respectively as mean values +/− standard deviation. (**B**) Control, RD and MADA cells left untreated (untreated) or after rapamycin treatment (rapamycin) were stained for Oct-1. Arrows indicate Oct-1 nuclear foci, arrowheads indicate recovered Oct-1 localization in the nucleoplasm. The pseudo-coloring of Oct-1 pictures obtained by using the Photoshop 7 color mapping function reveals the accumulation of Oct-1 in nuclear foci and at the periphery and recovery by rapamycin treatment in MADA. The intensity of fluorescence is represented on a pseudocolor scale (palette bar). The percentage of nuclei with Oct-1 mislocalized in nuclear clusters is reported in the graph as mean values +/− standard deviation. (**C**) Proximity Ligation Assay (PLA) between prelamin A and Oct-1. Control and MADA fibroblasts left untreated or treated with rapamycin, were labeled with anti-Oct-1 and anti-prelamin A (SC - 6214) antibodies and probed with Duolink (Sigma) detection reagents according to the manufacturer. Nuclei were counterstained with DAPI. The PLA signals (in red) were counted with the Duolink ImageTool software and the average number of spots in the nucleus per cell (200 cells per sample were counted) is presented in the graph. Statistically significant differences (p<0.05) are indicated by asterisks.

The inner nuclear lamina constituent LAP2alpha has been shown to be affected by prelamin A accumulation [24]. In MADA and RD cells, LAP2alpha was dramatically reduced in a percentage of nuclei and mislocalized into nuclear aggregates, while rapamycin treatment elicited rescue of protein localization (Fig. 3A). In laminopathic cells, we also noticed anomalous recruitment to the nuclear rim of the transcription factor Oct-1, implicated in stress response pathways (Fig. 3B). This effect could be attributed to competition with lamin B1, showing sequence homology with prelamin A, including the farnesylated and carboxymethylated C-terminus, and shown to be able to sequester Oct-1 [15, 34]. Oct-1 was also accumulated in intranuclear clusters in MADA cells (Fig. 3B). Thus, we wondered if reduction of farnesylated prelamin A levels might rescue Oct-1 localization in the nuclear interior. Rapamycin treatment caused partial import of Oct-1 in the nucleoplasm and complete removal of nucleoplasmic foci (Fig. 3B). Importantly, as shown by proximity ligation assay, Oct-1 was bound to prelamin A in MADA nuclei, with evident signals at the nuclear envelope (arrow in Fig. 3C) and in intranuclear foci (arrowhead in figure 3C). Rapamycin led to complete release of binding in MADA nuclei (Fig. 3C). Thus, entrapment of Oct-1 by prelamin A in MADA can account for its mislocalization and could be relevant to Oct-1 transactivation activity and cellular response to stress [35].

### Rescue of chromatin organization in rapamycin treated laminopathic cells

**Figure 4:**
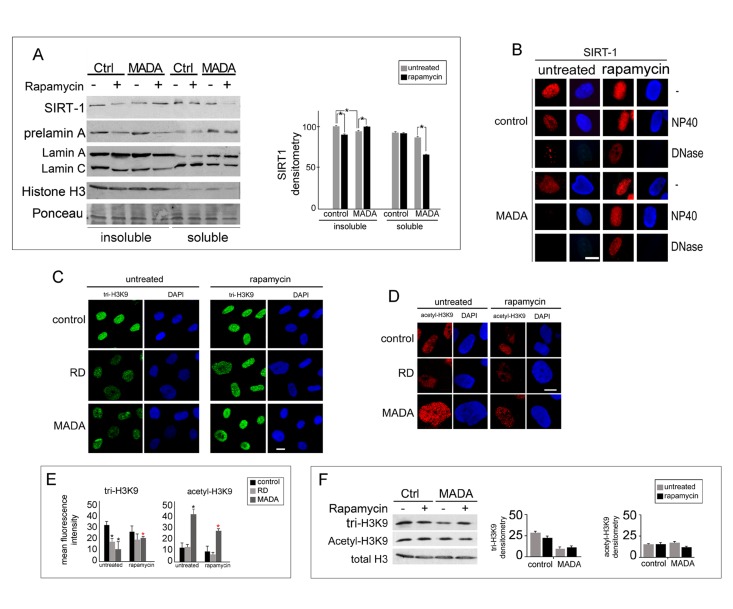
Chromatin-associated proteins affected in MADA are rescued by rapamycin (**A**) Western blot analysis of SIRT-1 in control and MADA lysates left untreated or after rapamycin treatment. The lysates were obtained in SDS buffer (insoluble) or in RIPA buffer (soluble) to evaluate the amount of SIRT-1 in different nuclear platforms; densitometric values of immunoblotted SIRT-1 bands are shown in the graph as means +/− standard deviation. Statistically significant differences (p<0.05) measured by the Mann-Whitney test are indicated by asterisks. (**B**) SIRT-1 staining in control or MADA cells left untreated (untreated) or subjected to rapamycin treatment (rapamycin). Non-extracted nuclei (−), nuclei subjected to detergent extraction (NP40) or to DNase treatment and high salt extraction (DNase) are shown. Nuclei were counterstained with DAPI. Scale bar, 10 μm. (**C**, **D**) Control, RD and MADA cells left untreated (untreated) or after rapamycin treatment (rapamycin) were stained for trimethyl-H3K9 (tri-H3K9) or acetyl-H3K9. Tri-H3K9 antibody labeling was revealed by FITC-conjugated secondary antibody (green), acetyl-H3K9 antibody labeling was revealed by TRITC-conjugated secondary antibody (red). (E) Tri-H3K9 and acetyl-H3K9 mean fluorescence intensity values measured by the NIS software in 200 nuclei are plotted. Statistically significant differences (p<0.05) are indicated by asterisks. Black asterisks indicate significance versus control cell cultures, red asterisks indicate significance versus the corresponding untreated sample. (**F**) Western blot analysis of trimethyl-H3K9 (tri-H3K9) and acetyl-H3K9 in control (ctrl) or MADA fibroblasts (MADA) before or after rapamycin treatment. Total H3 is reported as a loading control. The densitometric analysis of immunoblotted tri-H3K9 and acetyl-H3K9 bands is reported in the graph as mean values +/− standard deviation of the mean obtained in three different experiments. Statistically significant differences (p<0.05) measured by the Mann-Whitney test are indicated by asterisks.

Loss of peripheral heterochromatin is known to occur in RD and MADA cells, as indicated by altered histone methylation patterns and electron microscopy analysis [9, 19, 36]. A chromatin factor implicated in the regulation of H3K9 trimethylation is SIRT-1, a sirtuin required for the histone methyltransferase Suv39H1 stabilization and activity [37, 38]. SIRT-1 levels were reduced in the insoluble histone-containing fraction of MADA fibroblasts, while rapamycin elicited a SIRT-1 increase (comparable to controls) and reduction of the insoluble protein levels (Fig. 4A). In situ extraction of soluble proteins and DNA showed that the nuclear matrix-bound SIRT-1 increased in MADA after rapamycin treatment (Fig 4B). Moreover, we could observe recovery of trimethyl- H3K9 (tri-H3K9) distribution patterns and trimethylation levels in rapamycin-treated laminopathic fibroblasts (Fig. 4C), which indicated recovery of heterochromatin organization. Consistently, levels of acetyl-H3K9, which were slightly increased in MADA nuclei, were lowered after rapamycin treatment (Fig. 4D-F). Although changes in tri-H3K9 and acetyl-H3K9 amount were not statistically significant, the in situ analysis of H3K9 modifications showed an obvious increase in fluorescence intensity indicating recovery of conformational state. These results indicated that rapamycin might be effectively used to rescue the chromatin phenotype in laminopathic cells, due to its ability to trigger SIRT-1 increase and heterochromatin reorganization.

### Cell cycle dynamics is partially rescued by rapamycin in MADA

**Figure 5:**
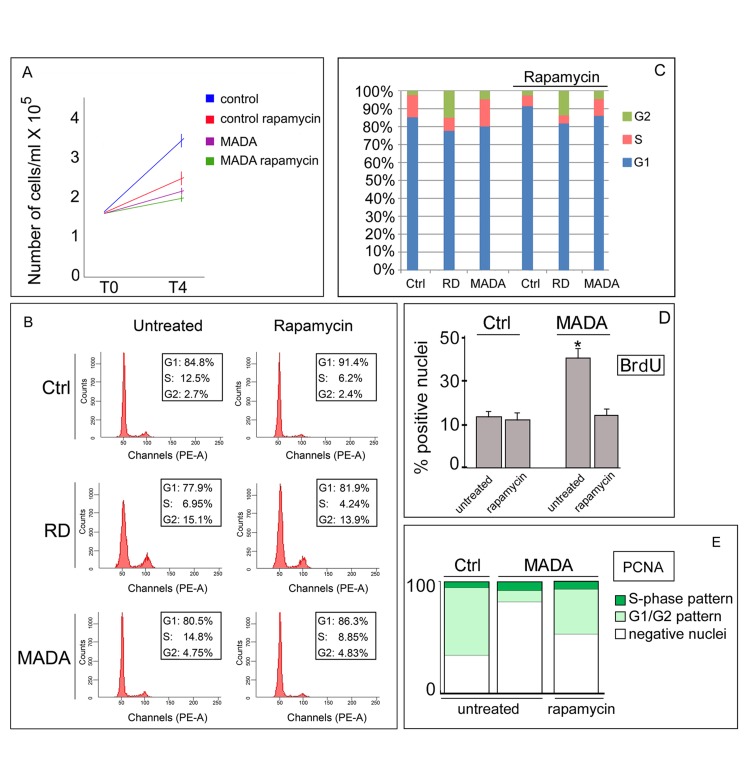
Cell cycle dynamics is partially rescued by rapamycin in MADA fibroblasts (**A**) Proliferation rate of control or MADA cells left untreated or treated with rapamycin. 1,45 × 10^5^ cells were plated at T0. After the treatment (T4), living cells were counted with trypan blue by a Neubauer camera. (**B**) Cell cycle analysis of cells left untreated or treated with rapamycin. The acquired FACS data were analyzed by ModFit LT software (Verity Software House, Inc.). Percentage of cells in cell cycle phases (G1, S, G2) is reported in the boxed areas within each panel. Ctrl, control. (**C**) Relative percentage of cells in each cell cycle phase before and after rapamycin treatment (rapamycin). (**D**) BrdU was incorporated into living fibroblasts for 4 hours and cells were fixed and subjected to IF staining using anti-BrdU antibody. The percentage of positive nuclei is indicated in the graph as mean value of three different counts obtained in different experiments. Data are means of three different counts +/− standard deviation of the mean. (**E**) Percentage of PCNA-positive MADA nuclei showing diverse staining patterns (S-phase, G1/G2 pattern). Subconfluent MADA fibroblasts left untreated (untreated) or treated with rapamycin (rapamycin) were immunolabeled for PCNA using anti-PCNA PC10 antibody. The number of nuclei with S-phase or G1/G2 staining pattern or negative for PCNA labeling was determined by counting 200 nuclei per sample.

Despite the lower proliferation rate observed in untreated MADA fibroblasts with respect to controls (Fig. 5A), FACS analysis (Fig. 5B and C) and BrdU incorporation into living MADA fibroblasts (Fig. 5D) showed an accumulation in S phase relative to controls, suggestive of a prolonged S phase [39], as previously reported in other MADA cells [40]. In fact, S-phase length was 2,4 fold longer in MADA (Table 1). Conversely, RD cells showed a reduced S phase fraction relative to controls (Fig. 5C, D and Table 1).

**Table 1:** S-phase and G2 length in RD and MADA cells before and after rapamycin treatment. S phase and G2 length were calculated using the formulas: T(S) = [Tc *ln(F(S) + 1)]/ln2 and T(G2) = [Tc *ln(F(G2) + 1)]/ln2, Tc = cell cycle length, *F(S)*= percentage of S phase cells, *F(G2)* = percentage of G2 cells [68].

	S phase	S phase (+rapamycin)	G2	G2 (+rapamycin)
**Control**	8 hr	4 hr	2 hr	1,4 hr
**RD**	7hr	4hr	14hr	13hr
**MADA**	19 hr	12hr	7 hr	7hr

Moreover, a significantly increased amount of G2 cells was observed in MADA as well as in RD (Fig. 5B and C). Rapamycin treatment reduced the S phase length in MADA cells and in the other cell cultures (Table 1), but did not affect the proportion of G2 cells or G2 length (Fig. 5B and C and Table 1). The evaluation of these data indicated partial rescue of cell cycle dynamics by rapamycin in MADA. To support this finding, we measured PCNA positivity of asynchronous fibroblast cultures before and after drug treatment. As expected, a much lower percentage of nuclei were positive for PCNA staining in untreated MADA cells relative to controls (Fig. 5E), indicating that a relevant percentage of cells had exited the cell cycle. In MADA, rapamycin effect was impressive, with rescue of PCNA positivity in 42% of nuclei even in high passage cells (Fig. 5E). The latter finding suggested that, although the doubling time of MADA fibroblasts was not significantly changed by rapamycin treatment (Fig. 5A), a significant proportion of cells re-entered the cell cycle following drug treatment (Fig. 5E). However, PCNA increase has been also linked to DNA damage repair, since PCNA is required for trans-lesion synthesis at double-stranded DNA breaks [41]. Moreover, slow progression through S phase may be due to DNA damage. Thus, we investigated the occurrence of DNA damage sites and their composition in laminopathic cells by labeling the phosphorylated histone μH2AX and 53BP1, two key constituents of the DDR machinery.

In MADA and RD nuclei, 53BP1 was severely reduced with respect to controls, but μH2AX foci were detectable (not shown). This situation could reflect an unrepairable DNA damage [40, 42], but it has been clearly linked to senescence, since increase of μH2AX in the absence of 53BP1 foci has been demonstrated in senescent cells even in the absence of DNA breaks [43, 44].

## DISCUSSION

The study here reported shows that rapamycin can counteract loss of factors involved in nuclear stability in MADA, leading to recovery of LAP2alpha and Oct-1 localization, release of aberrant prelamin A-Oct-1 binding, SIRT-1-dependent rescue of the chromatin phenotype and shortening of prolonged S-phase associated with improvement of cell cycle dynamics. Overall, rapamycin was not effective in RD cells, possibly due to the complete absence of mature lamin A, which most likely contributes to the aberrant cellular phenotype in RD. This observation implies that drug treatment in laminopathies that show defective lamin A processing must take into account several factors, including levels and functionality of mature lamin A.

Our data demonstrate that molecules that govern large scale chromatin arrangement, such as LAP2 alpha and SIRT1, are affected in MADA. In MADA fibroblasts, not only altered histone methylation and acetylation patterns are observed, but also delay in S-phase completion. Based on the latter observation it is reasonable to argue that the R527H *LMNA* mutation and its effects on nuclear matrix integral or interacting proteins might affect DNA synthesis. A delay in S-phase progression had been previously reported in other MADA cell cultures [40] and it has been recently demonstrated in cells depleted of lamin B1, which feature altered histone marks and heterochromatin organization [49]. On the other hand, while recovery of prolonged S-phase and PCNA increase could indicate rescue of cellular proliferation, population doubling time was slightly affected by drug treatment. Based on these observation, we suggest that the first effects of rapamycin treatment are Oct-1 and 53BP1 recruitment to damaged DNA sites and association with PCNA, which facilitate DNA repair and shorten S-phase, ultimately improving chromatin dynamics.

The effect here observed on Oct-1 localization has been recently ascribed to prelamin A accumulation and linked to impaired stress response and senescence induction [35]. Importantly, here we show in vivo binding of prelamin A and Oct-1 and sequestering of the transcription factor at the nuclear periphery and in intranuclear clusters in MADA cells. In that context, rapamycin treatment elicits complete release of binding, indicating functional improvement in patient cells. Thus, our study supports the view that slight reduction of prelamin A to non-toxic levels is sufficient to rescue aberrant localization of the transcription factor and may contribute to recovery of the pathological phenotype.

Several studies have shown that reduction of prelamin A levels either by molecular approaches [50] or drug treatment [8] improves the cellular phenotype in progeroid laminopathies. Here, we used a drug, rapamycin, which interferes with m-TOR inhibition of autophagy, thus triggering the autophagic pathway. In fact, autophagy is a physiological degradation mechanism, mostly aimed at scavenging damaged organelles, but also implied in the elimination of altered proteins [22, 29, 51]. Of note, rapamycin has been shown to reverse elevated mTORC1 signaling in lamin A/C-deficient mice, showing that a direct effect of the drug on the m-TOR pathway is beneficial not only in progeroid, but even in muscle laminopathies [52]. This could be attributed to the inhibition of geroconversion exerted by rapamycin, i.e. to its ability to delay the shift from cell cycle exit to senescence in cells [45, 46], [47], [53]. Thus, not only the rapamycin effects on prelamin A levels here reported, but also the direct or possibly lamin-mediated effects of the drug on mTOR activity, a master regulator of the aging process [48], indicate rapamycin as a powerful therapeutic tool for MADA and other progeroid diseases.

Thus, it is not surprising that rapamycin has been demonstrated to alleviate aging and extend lifespan in animal models [52, 54-56] and we have recently reported that this drug mimics a situation detectable in cells from centenarians through modulation of prelamin A forms and 53BP1 recruitment [4]. Further, SIRT-1 activity has been involved in mechanisms that might increase longevity, including caloric restriction. In this context, the effect here described of rapamycin on SIRT-1 recruitment to the nuclear matrix could be relevant not only to progeroid disorders, but also to the normal ageing processes.

The efficacy of rapamycin in Hutchinson-Gilford progeria cells accumulating the truncated prelamin A form called progerin has been reported [57] [31]. On the other hand, since our data here reported and recently published results [27] clearly show spontaneous degradation of non-farnesylated prelamin A through lysosomes, any drug treatment impairing protein farnesylation is expected to contribute to improvement of the cellular and clinical phenotype in laminopathies [1, 8, 58]. In this respect, some efficacy of ongoing clinical trials with statins and bisphosphonates [58, 59], which impair prelamin A farnesylation, might be related to the increased susceptibility of non-farnesylated prelamin A to lysosomal degradation. Our data can be matched to clinical and pharmacokinetic parameters to design comprehensive and efficient therapeutic strategies [58, 60-62] for MADA and other laminopathies.

## METHODS

### Cell cultures, transfection and treatments

Skin fibroblasts from RD and MADA were obtained from two newborn children carrying the 1085_1086 insT mutation in FACE1 gene and two adult patients carrying the R527H *LMNA* mutation, respectively. Control skin fibroblast cultures were obtained from skin biopsies of healthy patients (mean age 12) undergoing orthopedic surgery. A written consent had been obtained from patients or their families and all the local and EU ethical rules were applied. Cells were cultured in Dulbecco's modified Eagle's medium - High Glucose (DMEM-HG) supplemented with 20% Fetal Calf Serum (FCS) and antibiotics mix at 37°C and 5% CO_2_. The experiments were performed at passages 12-18. Human embryonic kidney HEK293 cells were cultured in DMEM-HG supplemented with 20% FCS at 37°C and 5% CO_2_. Transient transfections of HEK293 cells were performed by the calcium phosphate method.

Where indicated, cells were treated with 1 μM rapamycin for 8 or 24 hours (HEK293 cells) or 4 days (human skin fibroblasts). To block lysosomal or proteasomal activity, cells were respectively treated with 25 μM chloroquine or 10 μM MG132 for 8 or 24 hours. All the reagents were from Sigma, St. Louis, MO, U.S.A.

### Plasmids

FLAG-tagged plasmid containing rat wild-type prelamin A (LA-WT) has been previously described [63]. By means of the QuikChange strategy (Stratagene, La Jolla, CA, U.S.A), LA-WT cDNA was used as template to generate the following prelamin mutant forms: LA-C661M, encoding a unfarnesylated unprocessed prelamin A, LA-L647R, encoding a farnesylated, carboxy-methylated unprocessed prelamin A [64] and LA-R527H, encoding a mutant lamin A form associated with MADA. LA-S404A was used as non-phosphorylatable prelamin A mutant, LA-S404D as a phosphomimetic prelamin A mutant [27]. All the mutation primers used are described in Table 2.

**Table 2:** *LMNA* mutagenic primers used in this study.

Lamin A mutant	Forward mutagenic primer	Reverse mutagenic primer
R527H	5’ GGGAGCAGCCTTC**A**CACGGCTCTCATCA 3’	5’ TGATGAGAGCCGTG**T**GAAGGCTGCTCCC 3’
L647R	5’ CCGCTCCTACC**G**CCTGGGCAACTC 3’	5’ GAGTTGCCCAGG**C**GGTAGGAGCGG 3’
C661M	5’ CAGAGCTCCCAGAAC**ATG**AGCATCATGTAATC 3’	5’ GATTACATGATGCT**CAT**GTTCTGGGAGCTCTG 3’
S404A	5’ GCCGCGCCTCC**G**CCCACTCCTCCC 3’	5’ GGGAGGAGTGGG**C**GGAGGCGCGGC 3’
S404D	5’ GCCGCGCCTCC**GA**CCACTCCTCCC 3’	5’ GGGAGGAGTGG**TC**GGAGGCGCGGC 3’

### Antibodies

Antibodies employed for Western blot analysis or immunofluorescence labeling were: anti-lamin A/C (goat polyclonal, Santa Cruz SC-6215) and anti-prelamin A (goat polyclonal, Santa Cruz SC-6214); anti-farnesyl-prelamin A, (rabbit polyclonal, Diatheva 1188-2), raised against the last 15 aminoacids of the prelamin A sequence including the farnesylated cysteine residue but not the SIM sequence [65]; anti-prelamin A, (rabbit polyclonal, Diatheva 1188-1), raised against the last 18 aminoacids of the prelamin A sequence [65]; anti-LAP2alpha (rabbit polyclonal, [66]); anti-trimethyl-H3K9, rabbit polyclonal and anti-acetyl-H3K9, rabbit polyclonal (Upstate); anti-emerin, mouse monoclonal (Monosan); anti-LC3-B, rabbit polyclonal (Cell Signaling Technologies); anti- 53BP1 antibody (Cell Signaling); anti-gammaH2AX (Abcam); anti-Oct-1 (Santa Cruz); anti-actin, goat polyclonal (Santa Cruz), anti-phospho-Lamin A Ser404 rabbit polyclonal antibody [26].

### Preparation of whole and nuclear extracts and Western blot analysis

Whole cell lysates were prepared by the addition of RIPA buffer (20 mM Tris-HCl, pH 7.0, 1% Nonidet P-40, 150 mM NaCl, 10% glycerol, 10 mM EDTA, 20 mM sodium fluoride, 5 mM sodium pyrophosphate, 1 mM Na_3_VO_4_, 1 mM PMSF, 10 μg/ml leupeptin and 10 μg/ml pepstatin) at 4°C. Insoluble fraction was obtained by the addition of SDS-buffer, containing 20 mM Tris-HCl, pH 7.5, 1% SDS, 1 mM Na_3_VO_4_, 1 mM PMSF, 5% β–mercaptoethanol and protease inhibitors at 90°C.

Nuclei were purified as described [67]: 5 × 106 cells were lysed in 400 µl nuclear isolation buffer (10mM Tris-HCl, pH 7.8, 1% Nonidet P-40, 10mM μ-mercaptoethanol, 0.5mM phenylmethylsulfonyl fluoride, 1µg/ml aprotinin and leupeptin and NaF 5mM) for 8 min on ice. MilliQ water (400 µl) was then added to swell cells for 3 min. Cells were sheared by passages through a 22-gauge needle. Nuclei were recovered by centrifugation at 400 × *g* and 4°C for 6 min and washed once in 400 µl washing buffer (10 mM Tris-HCl, pH 7.4 and 2 mM MgCl2, plus inhibitors as describe above). The purity of the isolated nuclei was analyzed by detection of ß-tubulin (Sigma). Whole and nuclear lysates were diluted in Laemmli buffer, subjected to SDS-PAGE (8 %) and transferred to nitrocellulose membrane. Membranes were saturated with 4 % BSA and incubated with primary antibodies for 1 hour at room temperature. Secondary antibodies were used at 1:10000 dilution for 30 minutes. Immunoblotted bands were revealed by the Amersham ECL detection system. Intensity measurement was performed using a BioRad densitometer (GS 800) equipped with Quantity One Software.

### Gene expression analysis

Total RNA was isolated using Rneasy Mini Kit (Qiagen GmbH, Hilden, Germany) from confluent fibroblast cultures according to manufacturer instructions. RNA was reverse transcribed into cDNA using the High Capacity cDNA Reverse Transcription Kit (Applied Biosystems). *LMNA* or *ZMPSTE24* expression was evaluated by Real Time PCR, by amplifying 1 μg of cDNA with the TaqMan Gene Expression Assays (Applied Biosystems) on an Applied Biosystems StepOne termal cycler (Applied Biosystems). Probes and primers were all from Applied Biosystems, and were: GAPDH, assay ID Hs99999905_m1, LMNA, assay ID Hs00153462_m1*, ZMPSTE24, assay ID Hs00195298_m1*. For lamin C mRNA, HlmncFw CTGCGTACGGCTCTCATCA (exon 9) and HlmncRew GCGGCTACCACTCAC (lamin C-specific sequence), for prelamin A mRNA Hprelamin Fw ACTGGGGAAGAAGTGGCCAT (between exons 9-10) Hprelamin Rev GCTGCAGTGGGAGCCGT (between exons 11-10). The amplification protocol was: 50° for 2 min; 95°C for 10 min; 95°C for 15 s, 60°C for 1 min, for 40 cycles. The results were calculated as ratio between gene of interest and GAPDH reference gene and are expressed as the ratio between *ZMPSTE24* and *LMNA* mRNA expression. The experiments were performed in triplicate.

### FACS analysis

Control, MADA and RD samples were retrieved by trypsin detachment, fixed in 70% ethanol for 5 h, resuspended in PBS containing 5 g/ml RNase for 15 min at 37°C, washed in PBS and counter-stained with propidium iodide. The cells were then washed and analyzed by flow cytometry (FACSCanto II equipped with a 488 nm laser; 5000 events acquired).

BrdU detection. Human skin fibroblasts were plated onto coverslips. Cells were treated with rapamycin or vehicle as described above. At the end of treatment, cells were incubated with 10μM BrdU (Sigma) for 4 hours and then fixed in 70% ethanol. DNA was denatured by adding fresh 2N HCl and incorporated nucleotides were stained with anti-BrdU antibody (Becton Dickinson) according to manufacturer's instructions.

### Immunofluorescence and confocal microscopy

Human fibroblasts grown on coverslips were fixed with 4% paraformaldehyde at 4°C for 10 minutes and permeabilized with 0.15 % Triton X-100 for 5 minutes. Alternatively, cells were fixed with absolute methanol at -20°C. Non-specific binding was avoided by saturating samples with PBS/4% BSA. Coverslips were then incubated with primary antibodies overnight at 4°C following by secondary antibodies for 1 hour at room temperature. Anti-prelamin A SC-6214, anti-lamin A/C, anti-LAP2μ and anti-trimethyl H3K9 were used at 1:100 dilution. Anti-prelamin A 1188-2 was applied at 1:10 dilution. Anti-SIRT-1 was applied at 1:50 dilution, anti-Oct-1 at 1:100 dilution. Slides were mounted with an anti-fade reagent in glycerol and observed with a Nikon E 600 fluorescence microscope equipped with a digital camera. A Nikon Eclipse Ti microscope equipped with Confocal Unit A1-R was used to obtain optical sections in the z-axis at increments of 0.2 μm using a 60×, 1.3 NA objective and 488.0 nm laser lines to excite FITC (green).

### Proximity ligation assay (PLA)

Staining with primary antibodies was performed as described above for immunofluorescence microscopy using rabbit anti-Oct-1 (Santa Cruz Sc-232) and goat anti-prelamin A (Santa Cruz Sc-6214) antibodies. In-situ PLA was performed using the Duolink Fluorescence kit (Duolink® In Situ Red Starter Kit Goat/Rabbit, Sigma) according to manufacturer's protocol. Image acquisition was performed with a Zeiss Axiophot inverted fluorescence microscope using a digital camera and the Zeiss ZEN software. Signals were counted using the Duolink ImageTool software.

### Statistical analysis

Statistical analysis was performed using the Student's T test or the Mann-Whitney non-parametric test. Experiments were done in triplicate and differences were considered statistically significant for p<0.05.

## SUPPLEMENTAL FIGURE



## Abbreviations

MADA: Mandibuloacral Dysplasia
RD: Restrictive Dermopathy
Oct-1: octamer-binding transcription factor containing a POU domain with a homeobox subdomain
LAP2 alpha: lamina-associated polypeptide 2 alpha
SIRT1: sirtuin (silent mating type information regulation 2 homolog) 1
