# Supplementary material for: Rapamycin treatment of Mandibuloacral Dysplasia cells rescues localization of chromatin-associated proteins and cell cycle dynamics
Source: Aging (Albany NY). 2014 Jul 19;6(9):755–69. doi: 10.18632/aging.100680 (PMC4233654; doi:10.18632/aging.100680)
Supplement: Supplementary file 1 [file aging-06-755-s001.pdf]

SUPPLEMENTAL FIGURE

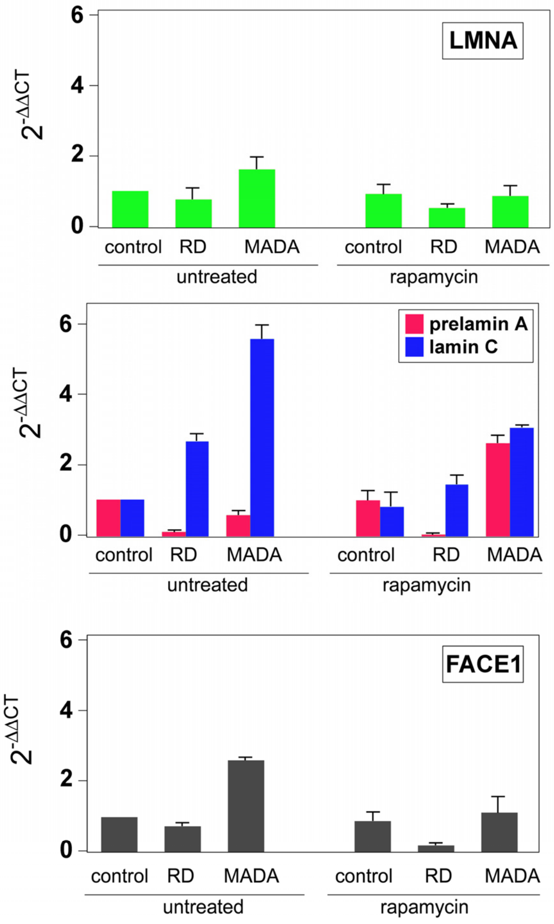

Supplemental Figure 1. Real-time RT-PCR analysis of *LMNA* and *FACE1* transcripts. Real-time RT-PCR analysis of *LMNA* and *FACE1* transcripts in untreated (untreated) and rapamycin-treated (rapamycin) control, RD and MADA fibroblasts. $2^{-\Delta\Delta CT}$ values are reported relative to untreated control samples.
